# New Relevant Evidence in Cholangiocarcinoma Biology and Characterization

**DOI:** 10.3390/cancers16244239

**Published:** 2024-12-19

**Authors:** Nunzia Porro, Elena Spínola-Lasso, Mirella Pastore, Alessandra Caligiuri, Luca di Tommaso, Fabio Marra, Alessandra Gentilini

**Affiliations:** 1 Department of Experimental and Clinical Medicine, University of Florence, 50139 Florence, Italy; nunzia.porro@unifi.it (N.P.); elena.spinolalasso@unifi.it (E.S.-L.); mirella.pastore@unifi.it (M.P.); alessandra.caligiuri@unifi.it (A.C.); fabio.marra@unifi.it (F.M.); 2 Department of Biomedical Sciences, Humanitas University, 20089 Milan, Italy; luca.di_tommaso@hunimed.eu; 3 IRCCS Humanitas Research Hospital, 20089 Milan, Italy

**Keywords:** cholangiocarcinoma, classification, tumor microenvironment, senescence, gut microbiota, long non-coding RNA, therapy

## Abstract

Among solid tumors, cholangiocarcinoma (CCA) emerges as one of the most difficult to eradicate. The silent and asymptomatic nature of this tumor, particularly in its early stages, as well as the high heterogeneity at genomic, epigenetic, and molecular levels delay the diagnosis, significantly compromising the efficacy of current therapeutic options and thus contributing to a dismal prognosis. Extensive research has been conducted on the molecular pathobiology of CCA, and recent advances have been made in the classification and characterization of new molecular targets. Both targeted therapy and immunotherapy have emerged as effective and safe strategies for various types of cancers, demonstrating potential benefits in advanced CCA. Furthermore, the deeper comprehension of the cellular and molecular components in the tumor microenvironment (TME) has opened up possibilities for new innovative treatment methods. This review discusses recent evidence in the characterization and molecular biology of CCA, highlighting novel possible druggable targets.

## Simple Summary

Cholangiocarcinoma (CCA) is a highly aggressive tumor with a poor prognosis. It represents about 15% of all primary liver tumors and about 3% of gastrointestinal malignancies, and its incidence is rising worldwide. Surgery constitutes the most optimal treatment for CCA, but only for early stages. The refractoriness to chemotherapy, the heterogeneity, and the silent and asymptomatic nature of this tumor contribute to its high mortality, which accounts for around 2% of all cancer-related fatalities globally each year. The molecular pathobiology of CCA has been the focus of numerous investigations in recent years, and new molecular targets have recently been classified and characterized. Both immunotherapy and targeted therapy have emerged as promising approaches for a variety of cancer types, including advanced CCA. Due to the desmoplastic features of CCA, progress in understanding the composition and functions of the tumor microenvironment has enabled the development of promising novel therapeutic approaches for CCA patients. This review has been created to describe the most important new evidence in the pathogenesis and characterization of cholangiocarcinoma, underlining the emerging topics in the cancer field.

## 1. Introduction

Cholangiocarcinoma (CCA) includes a group of heterogeneous cancers, resulting from the malignant transformation of biliary tree epithelial cells (cholangiocytes) [1,2,3,4,5]. The silent and asymptomatic nature of this tumor, particularly in its early stages, as well as the high heterogeneity of CCA at clinical, genomic, epigenetic, and molecular levels, delay the diagnosis, significantly compromising the efficacy of current therapeutic options and thus contributing to a dismal prognosis [1,2,3,4,5]. Both the incidence and mortality of CCA have risen worldwide, with CCA representing 3% of gastrointestinal malignancies and 2% of all cancer-related deaths [6].

Several risk factors have been related to cholangiocarcinogenesis, including choledochal cysts, cirrhosis, chronic biliary diseases such as Caroli Disease or primary sclerosing cholangitis (PSC), hepatitis B or C viruses (HBV, HCV), liver fluke parasites such as *Opisthorchis viverrini* and *Clonorchis sinensis*, and certain toxins (asbestos, dioxins, or nitrosamines). Furthermore, other common risk factors comprise alcoholic liver disease, diabetes, tobacco consumption, non-alcoholic fatty liver disease (NAFLD), and non-alcoholic steatohepatitis (NASH) [4,7]. All of these risk factors are characterized by a persistent inflammatory condition associated with an overproduction of pro-inflammatory cytokines that represents the typical background in which CCA arises.

A distinctive feature of CCA is the presence of an abundant desmoplastic stroma, composed of extracellular components and different cell types such as cancer-associated fibroblasts (CAFs), endothelial and lymphatic cells, tumor-associated macrophages (TAMs), tumor-associated neutrophils (TANs), regulatory T lymphocytes (Tregs), and natural killer (NK) cells, thus creating a complex tumor microenvironment (TME) that contributes to CCA development, progression, and treatment resistance [2,8].

CCA responds differently to immunotherapy for a distinct tumor immune microenvironment (TIME) and for immune escape mechanisms. To increase the effectiveness of immune checkpoint inhibitors (ICIs) and change these immunologically “cold” tumors into “hot” tumors, the TIME of these tumors has been examined and is now being assessed [9,10].

Currently, surgical resection is the only potentially curative treatment, but only 20–30% of patients meet the criteria. In addition, the 5-year overall survival rate (OS) is significantly low and is linked to a high rate of tumor recurrence. Deeper mechanistic insights into the pathogenesis of CCA are therefore required; the association between molecular patterns and clinicopathological characteristics that define different subtypes of CCA represent a major challenge for the identification of novel and effective therapeutic approaches [11].

## 2. Classification

### 2.1. Anatomical and Molecular Classification

Based on the anatomical location, CCA is classified as intrahepatic CCA (iCCA) and extrahepatic CCA (eCCA), the latter further distinguished into perihilar CCA (pCCA) and distal CCA (dCCA) [11]. iCCA is located proximally to the second-order bile ducts within the liver parenchyma, pCCA is localized between the second-order bile ducts and the insertion of the cystic duct into the common bile duct, and dCCA is confined to the common bile duct below cystic duct insertion [4,12].

**Figure 1:**
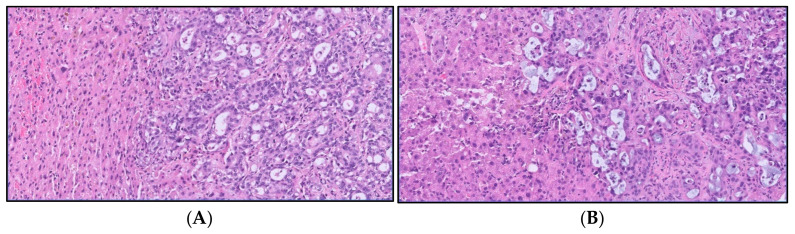
Representative histological images of CCA subtypes according to extent of the affected bile ducts. (**A**) A case of iCCA, small duct variant. The lesion is characterized by several homogeneous small glandular structures lacking overt cytological atypia and mucous production; the tumor border has an expansive pattern (H&E, 20×); (**B**) a case of iCCA, large duct variant. The lesion is characterized by irregularly shaped and distributed mucin-secreting glands, intermingled with fibrous tissue; the lesion has a moderate degree of cytological atypia and shows infiltrative margins (H&E, 20×).

According to the 5th WHO classification, iCCA comprises two distinct subtypes, based on the size of the affected bile ducts, the small duct type (SD-iCCA) and the large duct type (LD-iCCA), which display distinct clinicopathological characteristics in terms of risk factors, mutation profiles, survival, and response to chemotherapy (Table 1) [13,14]. Histologically, LD-iCCA displays a distinct glandular structure with mucin production linked to desmoplastic response. On the other hand, SD-iCCA is a heterogeneous tumor characterized by a ductular proliferation without mucin production (Figure 1). Better long-term outcomes are observed in the small duct type compared to the large duct type, partly due to its more aggressive nature [4].

In addition, a recent study has revealed that SD- and LD-iCCA are correlated with specific local immune profile patterns [14]. It has been found that most pathways in patients with SD-iCCA were downregulated, particularly those related to regulation, cell function, and adhesion, except for the complement, which showed increased activity. Furthermore, total tumor-infiltrating lymphocytes (TILs) were decreased in SD-iCCA, with a reduction in B cells/TILs, mast cells/TILs, and dendritic cells (DC)/TILs. On the contrary, LD-iCCA showed higher levels of total TILs and chemokine signaling, highlighting this subtype as potentially more responsive to immunotherapy [14].

A new classification independent from anatomical location has been recently proposed, identifying through a transcriptomic analysis two distinct subtypes in a cohort of 438 patients diagnosed with either dCCA, pCCA, or iCCA: the Mesenchymal and Immunosuppressive-C1 subtype and the Metabolic and Proliferative-C2 subtype. The Mesenchymal and Immunosuppressive-C1 subtype showed a stronger epithelial–mesenchymal transition (EMT), high signals of angiogenesis linked to tumor growth and invasiveness, and elevated hypoxia pathway activation. This subtype was partially categorized as “immunosuppressive” due to the strong presence of oncogenic signaling pathways that are linked to immune evasion, including IL-6/JAK/STAT3 and Wnt/β-catenin. A predominant infiltration of TAMs and Treg cells was observed, with low infiltration level of CD8^+^ T lymphocytes. Overexpression of genes encoding co-stimulators/co-inhibitors occurred, implying possible immune evasion mechanisms due to overexpression of immune checkpoints. On the contrary, the Metabolic and Proliferative-C2 subtype exhibited high mRNA levels of genes encoding MYC and E2F targets and activation of the G2/M checkpoint pathway, suggesting enriched proliferation-related features; it was defined by enriched metabolism-related hallmarks such as fatty acid, bile acid, and xenobiotic metabolism. In addition, genes encoding the peroxisome, an oxidative organelle involved in lipid metabolism, were found overexpressed. A predominant enrichment of PI3K/AKT/mTOR and mTORC1 oncogenic pathways was highlighted, which have been associated with ferroptosis resistance in cancer cells. The ferroptosis-related genes and ferroptosis signature score were dramatically lower in this subtype, demonstrating its ferroptosis-resistant feature. This subtype exhibited relatively high infiltration level of CD8^+^ T lymphocytes, CD4^+^ T memory resting cells, and B naïve cells [15].

### 2.2. Epigenetic Classification

Epigenetic dysregulation is involved in CCA pathogenesis. Recently, the characterization of the iCCA DNA methylome together with genomic, transcriptomic, and proteomic analyses have been useful for identifying distinct molecular subtypes, offering insights into potential therapeutic targets [7].

Particularly, Liao et al. presented a WGBS study comprising over 300 iCCA cases showing that iCCA can be classified into four DNA methylation subtypes (S1–S4), each of which exhibits different biological features and clinical outcomes. Global methylation loss was readily seen in S1–S3, with S2 showing the lowest methylation level among the four subtypes, whereas S4 demonstrated a methylation level comparable to that of normal controls. In addition, a pairwise analysis was conducted to define the gene expression profiles of each methylation subtype. The S1 group, composed of patients with isocitrate dehydrogenase 1 (IDH) mutation, was characterized by an upregulation of TGF-β signaling, ErbB signaling, and mTOR signaling, whereas S2 showed the highest enrichment of cell cycle/DNA replication pathways. The S3 group was KRAS-mutated and distinguished by significant interpatient variability in tumor immunity and a gene expression pattern linked with carbohydrate metabolism and cytoskeleton regulation/extracellular matrix (ECM) interaction. Patients with the S2 and S3 subtypes have the shortest survival among the four subtypes. The S4 subtype showed increased FGFR2 fusions/BAP1 mutations, and oxidative phosphorylation was found to be enriched [16].

The correlation between proteomics and DNA methylation was further investigated, and it was found that guanylate-binding protein-4 (GBP4), which belongs to a family of GTPases induced by interferon-gamma (IFN-γ), demonstrated the most significant prognostic implication and the most variable prevalence among methylation subtypes. Specifically, S2 and S3 showed the highest frequency of GBP4 demethylation. GBP4 plays a pivotal role in mediating type-I interferon responses and demonstrates a positive association with macrophage infiltration. These facts suggest an immune-activated microenvironment in iCCAs with demethylated GBP4, as highlighted by the enrichment of various innate/adaptive immune pathways, which inspires the adoption of immunotherapies for patients with demethylated GBP4 [17].

The main biological features and clinical outcome of each subgroup are summed up in Table 2 [16].

## 3. TIME

The TIME of CCA is characterized by a complex interaction between the numerous stromal and immune components. Particularly, emerging literature suggests that the CCA microenvironment is ‘immune cold’, lacking in cytotoxic immune cells (cytotoxic T lymphocytes (CTLs)) and NK cells, and rich in immunosuppressive elements, especially myeloid cells and regulatory Treg cells [18,19,20]. Additionally, tumor-infiltrating immune cells show elevated expression of immune checkpoint markers such CTLA4 and PD-1 and its ligand PD-L1, suggesting an immunosuppressive environment [20]. Elevated PD-L1 expression correlates with pTNM stage and reduced OS, and is inversely correlated with CD8^+^ TILs in CCA [21,22].

In the TIME, immune cells are usually characterized by a state of immunosuppression associated with the release of various cytokines such as IL6, CCL2, and CCL8 and immune checkpoint proteins (PDD1, LAG3, TIGIT, and CTLA4). This hampers effective antitumor immune responses in the presence of T cells, B cells, NK cells, and TAMs [23].

Two tumor subtypes, with distinct TIME and patient prognosis, were identified by scRNA-seq of 14 iCCA specimens based on the differential expression of the markers S100P and SPP1 (osteopontin) [24]. Moreover, a classification in three molecular subtypes, chromatin remodeling, metabolism, and chronic inflammation, was identified in 151 iCCAs by proteomics analysis, whole-exome sequencing, and scRNA-seq [25].

An elegant recent study developed immunocompetent CCA models with different genetic drivers and showed that each model presented a distinctive TIME and mutational landscape with different response to ICI. These results emphasize that numerous preclinical models are necessary to better understand therapeutic responses in distinct genetic subsets of human CCA [26].

A predictive response to immunotherapy for CCA patients can be obtained from the spatial distribution of the tertiary lymphoid structures (TLSs). TLSs are lymphoid organs resembling peripheral lymph nodes formed during chronic inflammations, including cancer [27]. High intratumoral TLSs score was associated with a positive prognosis and immunotherapy response of CCA and combined hepatocellular–cholangiocarcinoma (cHCC–CCA) patients [27,28]. In addition, it has been shown that, compared to TLS-poor iCCA, TLS-rich iCCA exhibited a noticeably higher vein- and tumor-infiltrating T cell density with more efficient immune responses [29].

### 3.1. TILs

Tumor-Infiltrating Lymphocytes (TILs) are immune cells involved in the immune response against cancer cells, composed of different subsets, including CD8^+^ cytotoxic T cells and CD20^+^ B cells [30]. In several recent studies, OS of CCA patients was positively correlated with the infiltration of CD8^+^ T cells and CD4^+^ T cells at the tumor site, in contrast to a negative correlation with the over infiltration of activated Regulatory T Cells (Tregs) [31].

A higher number of cells CD8^+^ and Cytotoxic T-Lymphocyte-Associated protein 4 (CTLA4)^+^ and cells expressing CD8 FOXP3 markers, recognized as TILs, was observed in patients with human epidermal growth factor receptor 2 (HER2)-amplified CCA. This increase suggests that modulation of the immune component in the TME may be induced by HER2 amplification that could be recognized as a potential therapeutic target [32].

Recently, the soluble form of T cell immunoglobulin and mucin domain-containing-3 (Tim-3) has been associated with T cell exhaustion and a decrease of CD8^+^ T cell response to PD-1 inhibition showing a potential role of this protein as an immune checkpoint target for CCA [33].

The role of B cells in CCA is not well-defined, but there is evidence that they may contribute to both pro-tumor and antitumor activities depending on their subtype and the cytokines they produce. Granzyme B^+^ B cells present a gene signature whose expression is directly proportional with a long-lasting time to recurrence in patients with iCCA undergoing liver transplantation, suggesting they may contribute to effective antitumor immunity. Moreover, Granzyme B^+^ B cells interact with various cell types, which exhibit strong interaction with tumor cells. Granzyme B is a serine protease typically found in cytotoxic T cells and NK cells, playing a crucial role in inducing apoptosis in target cells. The presence of Granzyme B^+^ B cells indicates an atypical function of B cells, traditionally associated with antibody production and humoral immunity. Their detection in the tumor microenvironment suggests that they may have other roles beyond conventional B cell functions, possibly in modulating immune responses against tumors [34].

### 3.2. Tregs

Higher levels of Tregs have been found in CCA tissues compared to normal liver tissue. Tregs prevent an efficient immune response against tumor cells and produce immunosuppressive cytokines such as IL-10 and TGF-β suppressing the activity of effector T cells (such as CD8^+^ T cells) and NK cells [31].

In a recent study, in iCCA tumor sites, two immune subpopulations including immunosuppressive CD39^+^Foxp3^+^CD4^+^ Tregs and immune exhausted-like CD39^+^PD-1^+^CD8^+^ T cells were identified correlating with a worse clinical prognosis. Interestingly, in this study, both clusters presented high levels of CD39, providing evidence that CD39 inhibition may represent a therapeutic target for both Treg and CD8^+^ T cells in iCCA patients [35]. Furthermore, it has been shown that low levels of Tregs were associated to FGFR2 fusion/rearrangement, which is correlated to low grade and tumor cell differentiation. Conversely, iCCA patients with higher levels of Tregs were associated with poor OS. This study suggests that the correlation of FGFR2 fusion/rearrangement with good prognosis in CCA may be due to a reduction of Treg infiltration [36].

### 3.3. NK Cells

NKs are components of the innate immune system that have demonstrated potent antitumor activity. These cells can recognize and eliminate cancer cells without prior antigen exposure [9]. NK cells can kill tumor cells directly through the release of cytotoxic granules (containing perforin and granzymes) and by producing cytokines [37]. A recent study highlighted how NK cells show antitumor effects against CCA in response to IL-2 and IL-18. Since IL-2/IL-18-induced NK cells kill CCA cells and express antigen-presenting cell-associated markers, they could act as antigen-presenting cells, exposing CCA-derived antigens to both CD4^+^ and CD8^+^ T cells and acting as a bridge between innate and adaptive immunity. Therefore, NK cells have demonstrated an important ability to act as effectors against CCA [38].

### 3.4. TAMs

The most common immunosuppressive myeloid cell type in the TIME of CCA is represented by TAMs, whose expression is associated with poor patient outcomes [39]. TAMs are characterized by an M2 phenotype, promoting tumor growth, angiogenesis, and metastasis and suppressing antitumor immune responses through the secretion of anti-inflammatory cytokines and growth factors such as IL-10 and TGF-β [36].

Recently, trefoil factor (TFF) proteins, already known to have tumorigenic activity in CCA, have been identified as a promoter of tumor M2 polarization of TAMs, characterized to the expression of CD163, CD206, and ARG-1 [37].

Gao et al. observed that patients with iCCA classified as highly inflammatory type showed a worse prognosis and a higher frequency of KRAS and TP53 mutations [40]. This subtype of CCA is characterized by an enrichment of the extracellular matrix remodeling and angiogenesis components and better response to ICI treatment, as it presents high immune checkpoint expression levels such as PD-1, PD-L1, and CTLA-4, and a greater infiltration of immunosuppressive cells such as M2-type macrophages. The level of M2 macrophage infiltration could serve as a prognostic biomarker, helping to stratify patients based on their response to therapies and overall outcomes [38].

Of interest, it has been demonstrated that immunoresponsive gene 1 (IRG1), gene encoding for the enzyme aconitate decarboxylase 1,remained at low levels in M2 macrophages. IRG1 overexpression could prevent macrophage polarization toward the M2 phenotype, also resulting in inhibition of proliferation, invasion, and migration of CCA cells, while IRG1 inhibition induced tumor progression by regulating STAT3 phosphorylation. These data indicate that IRG1 in TAMs may act as a potential therapeutic target in CCA [39].

## 4. CAFs

By releasing growth factors, cytokines, exosomes, and extracellular matrix proteins, CAFs are one of the most prevalent cell groups in the TME of CCA. Particularly, the interaction between cancer cells and CAFs contributes to the desmoplastic stroma characteristic of CCA, creating a physical barrier to immune cell infiltration and drug delivery. This interaction also enhances the aggressiveness of the tumor and its resistance to therapies [41]. By directly boosting cancer cell proliferation, promoting angiogenesis, and altering the TME, CAFs can induce carcinogenesis. These effects can be carried out by ligand–receptor interactions, which can be initiated by ECM components, growth factors, and inflammatory cytokines [42]. Furthermore, there is evidence that CAFs may exhibit immunosuppressive and other cancer-restrictive functions and can contribute significantly to metastasis [43].

The development of a CAF-enriched TME and tumor progression induces the activation of the TGF-β pathway. Recently, it was observed that in iCCA, the TGF-β-specific gene signature includes inflammatory mediators and the potent profibrotic gene Serpine1, which encodes for plasminogen activator inhibitor-1 (PAI-1) that is an important serine protease inhibitor expressed by tumor cells, endothelial cells, and CAFs [44].

Moreover, in vitro studies have shown that TGF-β is involved in the migration and invasion of CCA cells induced by factors released from Schwann cells. These cells are typically associated with peripheral nerve tissue [45], but in recent years, various studies have demonstrated their involvement in contributing to the progression of CCA by secreting growth factors and cytokines such as nerve growth factor (NGF). NGF induced the EMT process in CCA by binding to its high-affinity receptor tropomyosin receptor kinase A (TrkA) [46].

Another important growth factor for tumor progression is represented by platelet-derived growth factor-BB (PDGF-BB). Recently, it has been shown that the expression of PDGF-BB is higher in CCA patients than in healthy control. Moreover, this ligand and its receptor platelet-derived growth factor receptor-β (PDGFR-β) were more expressed in CCA patients with lymph node metastasis. In these patients, PDGF-BB expression was mainly localized in CAFs, and in vitro and in vivo studies have demonstrated that the PDGF-BB released by CAFs induced migration, invasion, and proliferation of CCA cells, formation of lymphatic vessels, and invasion of endothelial cells [47].

Several markers for CAFs within iCCA have been identified, such as αSMA, COL1A1, PDGFR-β, fibroblast associated protein (FAP), periostin, and fibroblast-specific protein 1 (FSP1; also known as S100A4) [48]. Long et al. observed an increase of a specific subclass of CAFs expressing FAP in both hepatocellular carcinoma (HCC) and iCCA. This subclass was found to be involved in angiogenesis and collagen biosynthetic processes. Notably, the infiltration of FAP^+^ CAFs was higher in CCA compared to HCC. Furthermore, spatial analysis of CCA tissues revealed co-localization of FAP-expressing CAFs with macrophages expressing secreted phosphoprotein 1 (SSP1), a macrophage subtype associated with shorter OS of patients [41]. In a recent study, a new marker of a specific subtype of CAFs, a glycan-binding protein LGALS1, has been identified. LGALS1 was drastically upregulated in human gastric cancer, and has an important role in iCCA progression by increasing the expression levels of C-C chemokine receptor type 2 (CCR2), ADAM15, a metalloproteinase involved in cell adhesion to ECM, and β-integrin, a transmembrane receptor that plays a crucial role in cell adhesion and migration [49].

Due to the development of advanced sequencing technologies, CAFs have emerged as a highly heterogeneous cell population. Single-cell RNA sequencing (scRNA-Seq) analysis has identified specific CAF subtypes with distinct transcriptomic profiles coexisting in the same tumor. In the context of iCCA, these include mostly inflammatory CAFs (iCAFs) and vascular CAFs (vCAFs), which are characterized by the upregulation of cytokines and growth factors, such as IL-1β and hepatocyte growth factor (HGF) [43]; myofibroblastic CAFs (myCAFs) characterized by the production of ECM components, including COL1A1 and hyaluronic acid; antigen-presenting CAFs (apCAFs), expressing major histocompatibility complex II (MHC-II) genes; EMT-like CAFs (eCAFs), expressing epithelial to mesenchymal markers; and lipo-fibroblasts (lCAFs), expressing genes involved in lipid metabolism [43,50,51].

Depending on the type of tumor, myCAF-expressed COL1A1 has been linked to both tumor-promoting and tumor-restraining activities [43]. While it is widely assumed that type I collagen promotes tumors through increased stiffness, correlating with a poor prognosis in CCA [52], it has been recently shown that COL1A1 seems not to contribute to iCCA growth [53,54]. In this regard, CAFs may be able to shift from a pro-tumor to an antitumor cell population by treatment approaches that target tumor-promoting CAF mediators while preserving the impact of tumor-suppressive mediators (such type I collagen) [53].

### ECM

The ECM, which acts as a repository for growth factors and other released molecules, facilitates the pro-tumoral interplay between stromal cells and tumor cells inside the desmoplastic stroma that defines iCCA [43]. The ECM in CCA is characterized by excessive deposition of fibrous proteins like collagen and hyaluronan, leading to a stiff and dense stroma. This desmoplastic reaction not only provides structural support to the tumor, but also creates physical barriers to drug penetration and immune cell infiltration. The ECM also interacts with cancer cells through signaling pathways that can influence their behavior, including migration, invasion, and survival [55].

LOX, which catalyzes collagen and elastin fibrillogenesis, induced tumor progression by several mechanisms, including cell invasion, EMT, and increase of stromal density. Moreover, all isoforms of this enzyme LOX, LOXL1–4, correlated with poor prognosis in CCA. It was shown that the pharmacological inhibition of these isoforms in combination with chemotherapy induced the reduction of tumor growth in a mouse model [56,57].

**Figure 2:**
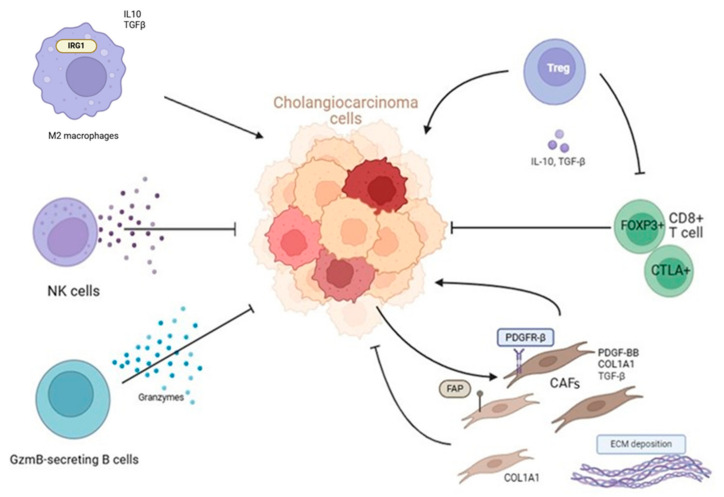
The tumor microenvironment in CCA. The CCA desmoplastic stroma is composed of extracellular components and different cell types that interact together as well as with components of the ECM and soluble factors within the TME to modulate CCA onset and progression. The immunosuppressive environment is mainly composed of TAMs, Tregs, and CAFs, which are mostly associated with worse outcomes. On the other side, cytotoxic CD8^+^ T cells (FOXP3^+^ and CTLA4^+^), GzmB-secreting B cells, and NK cells have antitumor effects.

The interactions between various cell types, including immune cells, stromal cells, and components of a dense ECM, within the CCA microenvironment are highly complex and contribute significantly to the aggressive nature of this cancer. These interactions facilitate tumor growth, immune evasion, and metastasis (Figure 2).

## 5. Cellular Senescence

Cellular senescence constitutes a state in which cells remain in a sustained and irreversible proliferation arrest. This state can be triggered by different stress types such as telomere shortening, oncogenic activation, irradiation, or genotoxic drugs. Senescent cells are generally characterized by enlarged cell and nuclear size, vacuolization, and granularity, increased senescence-associated (SA) β-galactosidase activity, and cell cycle arrest at G_1_ or G_2_/M phases. Proteins directly implicated in the regulation of the cell cycle such as p53/p21^WAF1/Cip1^ or p16^INK4A^ and phosphorylation of histone H2AX (γ-H2AX) are also considered senescence biomarkers. Although senescent cells are non-proliferating, they remain metabolically active and develop a highly varied and dynamic secretome consisting of proinflammatory cytokines, chemokines, growth factors, and matrix metalloproteinases (MMPs), collectively termed the senescence-associated secretory phenotype (SASP) [58,59]. Cellular senescence participates in physiological processes such as embryonic development or tissue regeneration and remodeling. However, during the process of aging, senescent cells accumulate and can contribute to age-related disorders including biliary liver damage, lung fibrosis, cardiovascular diseases and atherosclerosis, diabetes mellitus, osteoarthritis, or neurological disorders [58,60]. In the field of oncology, cellular senescence has been traditionally reported as a mechanism of tumor suppression by preventing the proliferation of damaged cells. Indeed, inducement of senescence represents a common mechanism of action of most treatment modalities such as chemotherapy (i.e., doxorubicin, cisplatin, and paclitaxel), radiotherapy, or targeted therapies (i.e., palbociclib and alisertib) [58]. More recently, the role of cellular senescence as a pro-tumorigenic driver has been observed in some specific contexts [61]. The persistence of senescent cells in the TME can contribute to tumor progression, angiogenesis, metastasis, and increased resistance to therapy via the release of immunosuppressive SASP [62,63]. Furthermore, studies in transgenic mouse models that allow the monitoring of senescent cells have shown that therapy-induced senescence is associated with the occurrence of side effects including bone marrow suppression, cardiac dysfunction, cancer recurrence, and decline in strength [61,63]. For these reasons, cellular senescence has become a promising target in cancer, leading to the development of therapies directed against senescent cells (senotherapy) [64].

Although both pro- and antitumoral properties of cellular senescence have been widely reported in the literature depending on the biological context, data about its implication in the development and progression of CCA are limited. In cholangiocytes, cellular senescence has been linked to the occurrence of cholangiopathies [65]. Particularly, in PSC [66], a premalignant disease characterized by chronic inflammation and fibrosis [67], increased cellular senescence has been observed in all disease stages, showing correlation with clinical severity and patient outcome [68]. Mechanistically, the induction of senescence causes stromal fibroblast activation via the release of SASP components, leading to liver fibrosis [66,69]. In contrast, overexpression of the polycomb-group proteins Bmi1 and EZH2, which are involved in the progression of human malignancies and particularly in CCA, have been reported to be decisive for bypassing senescence by a mechanism mediated by p16^INK4A^ gene repression in iCCA [70,71,72]. Telomere shortening constitutes one of the trigger factors of cellular senescence. In an in vitro model of eCCA, telomerase activity, responsible for telomere length maintenance, increased in response to interleukin-6 (IL-6) exposure, a pro-inflammatory cytokine that acts as a mitogenic in CCA. Increased telomerase activity delays senescence and, consequently, it promotes tumor proliferation [73]. The role of cancer-associated fibroblasts (CAFs), a major component of the TME, as tumor-promoting in iCCA has been suggested by Lan et al. [74]. They showed that elevated caveolin-1 expression in CAFs was linked to a poor prognosis. Moreover, based on previous works, they speculated that caveolin-1 mediated the development of a senescent phenotype in CAFs, which promoted iCCA proliferation through the release of SASP factors. However, this hypothesis needs further experimental verification.

Few studies have been published this year in regard to cellular senescence in CCA. Yang et al. developed a senescence-related gene signature that could be of prognostic value for iCCA patients [75]. Through co-expression and univariate Cox regression analyses, the authors identified 19 SA long non-coding RNAs (lncRNAs) that contributed to OS in a cohort consisting of 244 cases of iCCA. Based on the SA-lncRNAs expression, patients were stratified into two clusters that exhibited differential clinical and biological features. Cluster 1 showed a poorer outcome and worse prognosis than cluster 2. Furthermore, characteristics suitable with senescent phenotype such as upregulation of proteasome signaling, lipid, and amino acid metabolism, and higher stemness properties were observed in cluster 1. By the integration of different machine learning algorithms, an SA-lncRNA signature consisting of 11 lncRNAs was developed. Prognostic models were defined based on the signature and patients were classified in low-risk or high-risk groups. Molecular and clinical characteristics of the high-risk group were more consistent with cluster 1 showing poorer OS, higher immune suppression, and higher stem cell characteristics. High-risk iCCA samples also presented enrichment of senescence-related signaling pathways and higher SASP scores. Altogether, the data suggested that the SA-lncRNA signature exhibits characteristics of cellular senescence that potentially participate in the carcinogenesis of iCCA; however, it should be considered that data obtained in this work are mostly based on in silico analysis, so further verification in the clinical setting is needed. Conversely, the role of cellular senescence as a limiting factor to CCA tumorigenesis has been suggested by Ji and colleagues [76]. In their study, they generated a novel genetically transgenic mouse model of eCCA with promising value for eCCA research. This model was based on Pdx1-Cre-mediated PTEN conditional deletion in the epithelium and periductal glands of the extrahepatic biliary duct. The PTENL/L/Pdx1-Cre mice developed cholangitis, as demonstrated by an increase of epithelial proliferation, inflammatory cell infiltration, and fibrosis. The lesions gradually progressed to dysplasia, and eventually to invasive carcinoma. This murine model was further validated through expression analyses showing a more similar expression to human eCCA than human iCCA subsets. The lack of PTEN expression was associated with Akt activation, increased cell proliferation, accumulation of DNA damage, SA β-galactosidase staining, and increased EMT features. Moreover, when the deletion of p53, a tumor suppressor gene mutated in many CCA cases, was further introduced to generate PTEN^L/L^/p53^L/L^/Pdx1-Cre mice, the process of carcinogenesis was accelerated. Biliary epithelium hyperplasia was more marked and developed at younger ages, and mice survival was shorter than in p53 wild-type mice. Moreover, p53 loss abolished the presence of SA β-galactosidase-positive cells and enhanced proliferation. Overall, this work suggested the role of p53-mediated cellular senescence as a tumor limiting factor in eCCA development.

As mentioned before, cellular senescence mediates the antiproliferative activity of numerous antitumor drugs. In particular, its implication in the action of cannabidiol has been reported. Cannabidiol is a non-psychoactive ingredient of the cannabis plant with many pharmacological effects, including anti-inflammatory and anti-emetic. The anticancer properties of cannabidiol have been described in several malignancies including breast cancer, lung cancer, glioma, prostate cancer, and leukemia [77]. In CCA, its antitumor activity was first reported by Han’s group in 2022 [78]. Recently, the implication of cellular senescence in the mechanism of action of cannabidiol has been described both in vitro and in vivo in CCA [79]. The induction of senescence in response to the drug was revealed by G_1_ phase cell cycle blockade, increase of p21^WAF1/Cip1^ and p53 expression, and SA β-galactosidase staining in KKU-100 and KKU-213B cells. These findings were further supported by an increase of p21^WAF1/Cip1^ and β-galactosidase levels detected by immunochemistry in tumors from xenograft mouse models of CCA.

**Figure 3:**
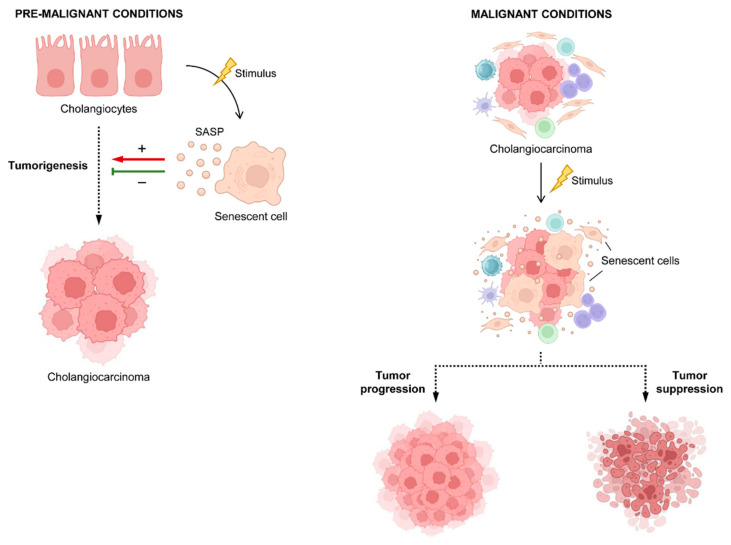
Dual role of cellular senescence in CCA. Under certain stimuli (i.e., telomere shortening, oncogenic activation, irradiation, or genotoxic drugs), cells can enter a state of sustained proliferation arrest, termed cellular senescence. In the context of CCA, cellular senescence constitutes a mechanism of tumor suppression by limiting the proliferation of pre-malignant lesions and tumors. Conversely, accumulation of senescent cells can contribute to tumor progression, metastasis, and increased resistance to therapy. SASP: senescent-associated secretory phenotype.

Studies addressing the implications of senescence in the context of CCA are limited. Some studies support that cellular senescence is involved in the pathophysiology of pre-malignant cholangiopathies. Regarding CCA, contradictory data exist about the implications of increased cellular senescence. Some authors argue the role of senescence as a tumor suppressor. Others suggest its association with cancer progression (Figure 3). Hence, research on the mechanisms regulating senescence in tumor cells, but also in the TME in CCA, is desirable and may clarify its value as a therapeutic target.

## 6. Non-Coding RNAs

### 6.1. Non-Coding RNAs: An Update

Non-coding RNAs (ncRNAs) are a heterogeneous class of RNAs without coding ability, and with diverse biological functions and mechanisms of action. In the last decades, an increasing number of studies has been dedicated to microRNAs (miRNAs) and, more recently, to lncRNAs and circular RNAs (circRNAs), three classes of ncRNAs that regulate main biological processes, such as chromatin remodeling, transcription, translation, and intracellular signaling, thus influencing cell behavior in physiological and pathological conditions. Due to their regulatory function, it is not surprising that a dysregulation of ncRNA expression/activity can play a crucial role in the pathogenesis of various diseases, including cancer [80].

### 6.2. LncRNAs

LncRNAs are transcripts longer than 200 nucleotides, with a 5′ cap and a 3′ tail, but lacking a complete reading frame, thus being unable to encode proteins. Depending on their localization, lncRNAs control diverse biological processes: in the nucleus they mainly modulate chromatin remodeling, RNA processing, and transcription, whereas in the cytoplasm they primarily regulate miRNAs and signaling pathways. The main mechanisms of lncRNA’s action include epigenetic changes, modulation of target genes through RNA binding proteins (RBPs) or other molecules, and competitive endogenous RNA (ceRNA) action, a process by which lncRNAs sponge miRNAs by annealing and inhibiting the interactions with their targets. Likewise, miRNAs and several lncRNAs were reported to be involved in CCA, and influence various cancer-related signaling pathways [80].

H19, the first identified lncRNA, has been recently recognized to promote liver cancer through multiple mechanisms. H19 was upregulated in NASH and cholestatic disorders, such as PBC and PSC, other than in cholestasis animal models [81]. In addition, overexpression of H19 was found in CCA cell lines and tissues, and correlated with tumor growth, TNM stage, post-surgical recurrence, and short survival time in CCA patients [82]. In CCA cells, H19 promoted proliferation, migration, and invasion, acting on different targets: it increased Bcl-2 expression via miR-216 downregulation [83], induced inflammatory mediators, particularly IL-6, sponging let-7a/let-7b [84], and enhanced EMT markers [85].

LINC00313 has been recently shown to play an oncogenic role as TGF-β signaling effector. Its overexpression in CCA was associated with KRAS and Tp53 mutations, and poor outcomes. LINC00313 gain of function in CCA cells favored colony formation in vitro and cancer growth in vivo. These effects were mediated by LINC00313 interaction with chromatin remodeling complexes and transcriptional induction of Wnt pathway-related genes [86].

LOXL1-AS1 lncRNA was also shown to promote CCA progression. It was overexpressed in CCA cells and tissues, and correlated with TNM stage, lymph node metastasis, and bad prognosis in CCA patients. In CCA cells, LOXL1-AS1 modulated proliferation, apoptosis, migration, and invasion. Mechanistically, high levels of LOXL1-AS1 led to a reduced ubiquitination and degradation of JAK2, and a consequent increase in the JAK2/STAT3 pathway. The transcription factor YY1 was identified as an upstream regulator of LOXL1-AS1, involved in CCA progression [87,88].

CCAT1 lncRNA was found to play a crucial role in CCA drug resistance. In particular, CCAT1 expression has been linked to erlotinib reduced sensitivity in CCA, induced by a positive regulation of EMT. This effect was mediated by CCAT1 modulation of miR-181a-5p/ROCK2 axis [89].

Conversely, Maternal Expressed Gene 3 (MEG3) lncRNA has been demonstrated to function as a tumor suppressor in CCA. MEG3 was markedly downregulated in CCA tissues in association with TNM stage, lymph node metastasis, and short survival time, being considered an independent predictor of bad prognosis in CCA patients. In CCA cells, low levels of MEG3 correlated with increased proliferation, invasion, and EMT, whereas MEG3 overexpression resulted in reduced tumor cell growth in vitro and in vivo [90]. Mechanistically, MEG3 acted as tumor suppressor in CCA through polycomb repressive complex 1 and by targeting miR-361–5p/TRAF3 axis [91].

LINC00844 is another recently recognized tumor suppressor in CCA. It was lowly expressed in CCA tissues and cell lines, and its upregulation in CCA cells resulted in decreased proliferation, migration, and invasion. These effects were mediated by the inhibition of miR-19a-5p, a LINC00844 direct target. Notably, LINC00844 expression in CCA patients was inversely correlated with miR-19a-5p levels. Moreover, low LINC00844 and high miR-19a-5p were predictive of poor prognosis, thus indicating their potential value as prognostic markers in CCA [92].

### 6.3. CircRNAs

CircRNAs, the most recently identified ncRNAs, are RNA molecules hundreds or even thousands of nucleotides long with 5′ and 3′ terminations covalently closed, to form a circular structure, which makes them stable and resistant to degrading enzymes. Due to these properties, circRNAs are particularly suited to be secreted and transported in extracellular vesicles, thus targeting different cells. Similar to lncRNAs, circRNAs exert their regulatory functions through multiple mechanisms, including miRNA sponging. As in other tumors, a number of dysregulated circRNAs have been identified in CCA, targeting multiple cancer-related pathways [93].

An example is represented by circ 0007534, which was very recently shown to be overexpressed in CCA tissues and cells, and associated with lymph node metastasis and poor outcomes in CCA patients. Its downregulation in CCA cells resulted in reduced proliferation, motility, stemness, and resistance to anoikis in vitro, and reduced tumor growth and metastasis in vivo. Interestingly, circ 0007534 upregulation was ascribed to positive feedback between this transcript and its parental gene, DDX42. Indeed, by binding to the DDX3X RBP, circ 0007534 formed a protein–RNA complex that stabilized DDX42 mRNA, increasing the expression of its product [94].

CircPCNXL2 has been involved in iCCA progression. It was found to be highly expressed in iCCA tissues and cells, and to induce tumor growth and metastasis in vitro and in vivo, and resistance to trametinib in vivo. These effects were mediated by a direct interaction of circPCNXL2 with serine-threonine kinase receptor-associated protein (STRAP) and the consequent activation of MEK1/2/ERK signaling, but also by the modulation of mir-766-3p/serine/arginine splicing factor 1 (SRSF1) axis [95].

### 6.4. Non-Coding RNAs as Potential Biomarkers and Therapeutic Targets

Increasing studies are demonstrating that aberrantly expressed ncRNAs have a critical role in CCA development and progression. A high tissue/cell specificity and a good correlation with clinical pathological parameters renders them potential diagnostic and prognostic tools for CCA. In addition, these molecules can be easily isolated and analyzed from tissues and body fluids (plasma, bile, and urine), which contain ncRNAs inside extracellular vesicles, as cargo. Currently, the diagnostic significance of ncRNAs in liquid biopsies, as a non-invasive procedure, is attracting increasing interest [96]. A preliminary study comparing healthy individuals, *Opistorchis viverrini* infected (asymptomatic) subjects, and CCA patients found plasma concentrations of miRNAs (cell free miRNA) were significantly higher in CCA, with high specificity and selectivity. Cell-free DNA content was also determined, with similar results. Interestingly, when the two indicators were matched, specificity and selectivity increased, making these findings encouraging and opening the way for further investigations [97]. Moreover, a better understanding of the mechanisms by which dysregulated ncRNAs affect CCA may help to manage patients and develop new therapies directed to ncRNAs or their targets.

## 7. Microbiota

Microorganisms that live inside individuals have a significant impact on physiology affecting both health and disease conditions, including cancer. Recent studies have shown that host microorganisms and their genes (microbiome) are implicated in the resistance of cancer patients to immunotherapy (IT) [98], by modulating immune checkpoint inhibitor efficacy [99]. In particular, the gut microbiome predicts responses to immunotherapy and prognosis, as different gut microbial patterns can be used to identify cancer patients from healthy individuals and responders from non-responders in a number of cancer cohorts treated with IT [100,101].

The gut microbiome influences the response to cancer therapy also by uncovering potent impacts on antitumor immunity through affecting the activity of primary and secondary lymphoid tissues, through microbial metabolic effects, and antigenic mimicry with tumor cells [99].

In addition to the gut microbiota, another niche of microorganisms that affect cancer development is the intratumoral microbiome. The TME presents a favorable setting for the growth of microorganisms, and for more than a century, these organisms have been found inside the tumors of cancer patients [98]. Typically, intracellular microorganisms infiltrate cancer, stromal, and immune cells inside the TME, and their main effect seem to inhibit local antitumor immunity [99,102,103,104,105,106]. Furthermore, tissue-resident microorganisms have been described to have mutagenic effects caused by released toxins [107,108,109,110], to affect chemoresistance [111], cancer proliferation, and metastasis [105,112,113], and to own pro-tumorogenic inflammatory activity [114,115,116,117].

Alterations of gut microbiota components have also been observed in CCA [118,119], especially in CCA-induced *Opistorchis viverrini* (OV) [120]. It has been shown that OV infection and dysbiosis in the intestines, bile, and tumors worsen cholangiocyte chronic inflammation and alter bile acid metabolism, which may contribute to the development of CCA [121].

Deng et al. suggested a gut microbiome signature based on eight genes that can differentiate CCA patients from healthy individuals [122], while Zhang et al. proposed a gut predictive model based on the pattern of three genera (B-F-R) for CCA early diagnosis [118] (Table 3).

In addition, four genera were found to be higher in iCCA patients than in healthy individuals or in patients with HCC. Interestingly, patients with vascular invasion had higher amounts of the family Ruminococcaceae, higher levels of plasma IL-4 and six conjugated bile acids, and lower levels of plasma IL-6 and chenodeoxycholic acid in comparison to patients with iCCA without vascular invasion. Thus, the authors identified gut microbiota, BAs, and inflammatory cytokines as indicators for the diagnosis of iCCA and the prediction of vascular invasion in iCCA patients [123].

A recent large-scale genome-wide association study (GWAS) using two-sample Mendelian randomization (MR approach) highlighted some gut microbiota taxa as predictive of a minor risk of CCA development. While elevated genetically predicted amount of the family *Veillonellaceae*, order *Enterobacteriales*, genus *Alistipes*, and phylum *Firmicutes* was linked to a lower risk of extrahepatic CCA, higher amounts of the genera *Collinsella*, *Eisenbergiella*, *Anaerostipes*, *Paraprevotella*, *Parasutterella*, and phylum *Verrucomicrobia* were linked to a decreased risk of iCCA [124].

Zhang et al. reported dysbiosis in feces and plasma of iCCA patients by 16S analysis. They also showed that plasma derived from iCCA patients was enriched in the metabolite glutamine that positively correlated with *Escherichia-Shigella* and *Subdoligranulum* [125] (Table 3). This finding is relevant, as glutamine metabolism is known to be involved in cancer invasion, growth, and therapeutic resistance [127].

A clinical trial called MICROBILIO(NCT04391426) that started in 2020 has the aim to characterize the microbiota and its alterations using the 16S rRNA sequencing method in bile samples of CCA patients (case group) and of living liver transplantation donors (control group) [128].

The CCA tumor microbiome has been investigated just recently. Xin et al. analyzed 121 CCA tissues and 89 paired nontumoral tissues for the tumor microbiome and found differences among bacteria composition (Table 3). Therefore, iCCA tumors could be differentiated from adjacent nontumoral liver tissue by their microbiota, which also showed a higher diversity of microbiomes. In addition, the authors showed that higher tumor microbial alpha diversity predicted shorter recurrence-free survival (RFS), and OS and was linked to lymph node metastases. A microbiological risk score, using the Lasso regression model to predict OS in iCCA, was developed [126].

### Probiotics

Probiotics are microorganisms that helps the host to restore microbial dysbiosis, maintaining intestinal microbial balance and inhibiting the colonization of harmful bacteria [7].

In 2024, a study was published on the role of extracts from the probiotics *Lacticasei bacillus rhamnosus* and *Staphylococcus simulans*, respectively, rhamnosin and lysostaphin, in CCA cell lines. In both parental and gemcitabine-resistant cells, a combination of the two bacteriocins suppressed growth and increased cell death more potently than the single agent, in part by upregulating the expression of the proapoptotic BAX, caspase-3, -8, and -9 genes.

As probiotics are known to affect cancer patients’ immune responses, some clinical trials have focused on gut microbiome modulation in cancer immunotherapy [129]. A clinical trial was conducted in liver cancer patients undergoing anti-PD-1 therapy, who received treatment with *Lactobacillus rhamnosus* Probio-M9 once a day during the whole treatment (NCT05032014). The trial was closed on December 2023, but there is no news on the success of the treatment.

However, up to now, there is no evidence showing that probiotics can affect CCA gut microbiota, and therefore, further investigations will be necessary to better understand the roles of these microorganisms in CCA.

## 8. Therapies

Therapeutic options for CCA are limited and they mostly include surgical resection, when diagnosed early, and systemic chemotherapy. According to EASL-ILCA Clinical Practice Guidelines, patients with unresectable CCA should be treated with GemCis, as first-line chemotherapy, with the addition of durvalumab. FOLFOX chemotherapy or ivosidenib for those with IDH1 mutations, FGFR inhibitors for those with FGFR2 fusions or rearrangements, and immunotherapy are recommended as second-line therapy [4].

Liver transplantation could be only considered for early stage iCCA (<3 cm), which arises in a cirrhotic background. Transplantation is indeed still contraindicated for iCCA due to microvascular invasion and poor tumor differentiation that leads to high tumor recurrence. However, emerging evidence proposes this approach as a possible strategy for small iCCA [4].

Although the high heterogeneity of CCA represents a limitation for successful universal treatments, it also opens new opportunities for personalized therapies. Remarkable progress in the field of CCA therapy has indeed paved the way to new potential therapeutic approaches, with targeted therapies and immunotherapy beginning to show benefits in improving patients survival rates [6].

### 8.1. Targeted Therapy

Whole and targeted DNA sequencing studies have outlined the genomic complexity of CCA tumors, identifying the prevalent druggable gene mutations that affect key signaling pathways (Table 4) [1,6,130,131].

#### 8.1.1. FGFR Inhibitors

The Food and Drug Administration (FDA) has currently approved three FGFR inhibitors for previously treated, unresectable, locally advanced, or metastatic CCA: pemigatinib, futibatinib, and infigratinib [132,133,134].

Approval of pemigatinib, a selective inhibitor of FGFR 1, 2, and 3, was based on FIGHT-202 (NCT02924376), a multicenter open-label clinical trial that demonstrated a clinically stable response and prolonged OS in patients with previously treated, locally advanced, or metastatic CCA with a FGFR2 gene fusion or other rearrangement [135,136]. Following these results, a phase III FIGHT-302 trial (NCT03656536) is currently underway, investigating the safety and efficacy of pemigatinib as a first-line treatment for patients with unresectable or metastatic CCA with FGFR2 fusion or rearrangement versus the current first-line systemic therapy of gemcitabine plus cisplatin (GEMCIS).

Another inhibitor of FGFR 1, 2, and 3 is infigratinib, which showed promising clinical activity and manageable adverse effects in previously treated patients with locally advanced or metastatic CCA harboring FGFR2 gene fusions or rearrangements [137]. Infigratinib received FDA approval following the results of a phase II clinical trial (NCT02150967) of 122 patients that demonstrated a 23.1% objective response rate (ORR) in treatment-resistant CCA patients with FGFR2 fusions or rearrangements [138]. Its efficacy has been further confirmed in the phase III PROOF 301 (NCT03773302) trial, which defined a new protocol for a chemotherapy-free, first-line FGFR2-targeted therapy [139].

However, a limitation of FGFR inhibitors is the development of therapeutic resistance, which can limit the effectiveness of these agents. De novo alterations in the FGFR2 kinase binding domain have been described as a mechanism of acquired resistance. Furthermore, MAPK pathway alterations seem to also be involved and thus, targeting these co-alterations could be a potential strategy to overcome acquired resistance to FGFR inhibitors [140]. Literature data have indeed highlighted the potential clinical utility of FGFR-targeting agents in combination with BRAF and MEK inhibitors as a promising strategy in metastatic tumors with FGFR2 pathway activation [141,142].

Futibatinib, an irreversible inhibitor of FGFR 1–4, has shown potent preclinical activity against acquired resistance [143,144]. FOENIX-CCA2, a phase II study, demonstrated a 42% ORR in 43 of 103 patients who received futibatinib, with a median progression-free survival (PFS) of 9 months, as well as tolerable adverse effects. Following these results, FOENIX-CCA4 (NCT05727176), an open-label, multinational, phase II study, aims at confirming the clinical benefit of futibatinib and evaluating the safety and efficacy in previously treated CCA harboring FGFR2 gene fusions and other rearrangements.

Additional next-generation inhibitors, such as the FGFR2-selective inhibitor lirafugratinib (RLY-4008) (NCT04526106) and the multikinase inhibitor tinengotinib (NCT04919642, NCT05948475), are currently under investigation to assess their efficacy in overcoming resistance to prior FGFR inhibitors [145,146]. RLY-4008 is a highly selective FGFR2 inhibitor that has activity against primary FGFR2 alterations and common resistance mutations. This agent demonstrated encouraging clinical activity in a first-in-human study involving 35 patients with FGFR2-altered CCA, with >10% tumor shrinkage observed in 56% of patients with CCA previously treated with an FGFR inhibitor [39,145,147].

Other FGFR inhibitors under investigation in patients with CCA include the pan-FGFR inhibitors erdafitinib (NCT02699606), KIN-3248 (NCT05242822), and ICP-192 (NCT05678270, selectively inhibits FGFR 1, 2, 3, and 4 activities irreversibly by covalent binding), the multikinase inhibitor derazantinib (NCT03230318), the bivalent FGFR1–3 inhibitor tasurgratinib (E7090, which blocks FGF–FGFR interactions; NCT04238715), and the FGFR1–3 inhibitor HMPL-453 (NCT04353375). Moreover, a phase II trial is investigating the combined treatment of atezolizumab (targeting the immune checkpoint protein PD-L1) and derazantinib in patients with advanced iCCA with FGFR2 fusions/rearrangements (NCT05174650) [39].

#### 8.1.2. IDH1 Inhibitors

IDH1 variations occur in around 20% of patients with iCCAs and 0.8% in pCCAs and dCCAs [148]. The mutations of IDH1 can lead to abnormal DNA methylation, resulting, in turn, in uncontrolled cell proliferation.

Ivosidenib, a selective inhibitor of mutant IDH1, was approved by the FDA in 2021 for the treatment of locally advanced unresectable or metastatic iCCA [149], since it has been shown to be safe and well-tolerated. The phase III ClarIDHy trial proved that patients with IDH1-mutant CCA who received ivosidenib had significantly higher median PFS and OS if compared to placebo [150]. Particularly, the median PFS improved to 2.7 months for the ivosidenib group compared to 1.4 months for the placebo group. Moreover, its favorable PK/PD profile supports the 2-hydroxyglutarate (2-HG) lowering mechanism of action and provides good outcomes for patients receiving treatment for advanced mIDH1 CCA [151,152].

However, secondary IDH1 mutations and IDH1/2 isoform switches can arise, leading to ivosidenib resistance after long-term IDH1 inhibitor treatment [153]. For this reason, additional IDH1 inhibitors are under investigation in patients with CCA, including olutasidenib (FT-2102; NCT03684811), LY3410738 (NCT04521686), AB-218 (NCT05814536), and HMPL-306 (NCT04762602). In addition, since IDH1 mutations can affect the mechanism of double-stranded DNA damage repair [154], several ongoing trials are also evaluating in mIDH1-CCA patients the efficacy of poly(ADP-ribose) polymerase (PARP) inhibitors such as olaparib (NCT03212274), olaparib plus the ATR inhibitor ceralasertib (NCT03878095), and olaparib plus durvalumab (NCT03991832).

#### 8.1.3. KRAS-RAF-MEK-ERK Inhibitors

Proto-oncogene KRAS-activating mutations are common in CCA and are associated with unfavorable PFS and OS. KRAS activation upregulates signaling via downstream pathways, including the RAF–MEK–ERK (MAPK) pathway. Thus, MEK inhibition may be a potential treatment strategy for CCA patients with KRAS mutation [39].

The ABC-04 phase Ib trial has shown that the combination of selumetinib (MEK1/2 inhibitor), gemcitabine, and cisplatin is safe and can be used in patients with advanced biliary tract cancer. However, its role as an effective therapy will be determined by further randomized phase II and III trials (NCT02151084) [155]. An ongoing open-label, multicenter phase 1b/2a study is instead evaluating safety, pharmacokinetics (PK), pharmacodynamics (PD), and efficacy of GNS561 (a new autophagy inhibitor), in combination with trametinib (MEK inhibitor) in advanced KRAS-mutated CCA after failure of standard-of-care first line therapy (NCT05874414).

Up to 5% of biliary tract tumors (BTCs), mainly iCCAs, have BRAF V600E mutations that are linked to a reduced OS time [156]. The ROAR (Rare Oncology Agnostic Research) trial is a multicenter, open-label, and phase II basket trial designed to evaluate the efficacy and safety of dabrafenib (oral BRAF inhibitor) plus trametinib (oral MEK inhibitor) for patients with rare cancers harboring BRAF V600E mutations. Particularly, in the BTC cohort of the phase II ROAR basket trial, the treatment with the BRAF inhibitor dabrafenib in combination with the MEK inhibitor trametinib resulted in an ORR of 47%, a median PFS of 9.0 months, and median OS of 14 months [39,157].

An ongoing phase I clinical trial (NCT05501912) is evaluating the efficacy and anti-cancer activity of ABM-1310, a highly potent and selective BRAF inhibitor, in patients with BRAF V600-mutant advanced solid tumors, including CCA. Moreover, the phase II ComboMATCH treatment trial (NCT05564403) is evaluating whether binimetinib and mFOLFOX6 combination therapy (modified leucovorin, fluorouracil, and oxaliplatin) improves OS compared to mFOLFOX6 alone in patients with advanced/recurrent BTC and with alterations in the RAS/RAF/MEK/ERK pathway.

#### 8.1.4. HER2 Alterations

HER2, belonging to the ErbB family of receptor tyrosine kinases, is overexpressed more frequently in pCCAs and dCCAs (17.4%) than in iCCAs (4.8%) and could represent a plausible target in the CCA treatment. In the phase II MyPathway basket trial, patients with HER2-amplified and/or overexpressing BTCs received trastuzumab in combination with pertuzumab (HER2 monoclonal antibodies), resulting in an ORR of 23% and a median PFS of 4.0 months [158]. Another phase II trial evaluated the efficacy of combination folinic acid, 5-fluorouracil, and oxaliplatin (FOLFOX) chemotherapy plus trastuzumab in the second line setting in 34 patients with HER2-positive BTCs [159].

A phase II trial, the DESTINY-PanTumor02 Phase II Trial (NCT04482309), highlighted durable clinical benefit, meaningful survival outcomes, and safety in pre-treated patients with HER2-expressing tumors after the administration of the antibody–drug conjugate trastuzumab deruxtecan (T-DXd) [160]. T-DXd is being further explored in patients with BTCs in an ongoing phase II trial (NCT04482309).

Additionally, the oral, irreversible, pan-ErbB tyrosine kinase inhibitor neratinib has been evaluated in patients with previously treated HER2-mutant CCA as part of the SUMMIT basket trial. Results from the phase II BTC cohort of this trial, which included 11 patients with CCAs, demonstrated an ORR of 16% in all 25 patients evaluated, with a median PFS of 2.8 months and median OS of 5.4 months. A recent clinical trial (NCT06519110) is assessing the efficacy of neratinib monotherapy in treating advanced solid tumors with HER2 mutations.

The novel bispecific anti-HER2 antibody zanidatamab, which targets the same epitopes as trastuzumab and pertuzumab, showed positive results, with tumors shrinking by 30% or more of their original size in 41% of patients of the study [161]. This agent is being further evaluated in studies including patients with CCAs and other BTCs (NCT04466891 and NCT03929666). Other ongoing therapeutic strategies evaluated in trials involving patients with HER2-overexpressing CCAs include a novel anti-HER2 monoclonal antibody IAH0968 in combination with gemcitabine and cisplatin (NCT05991518), a novel anti-CD47/HER2 bispecific antibody IMM2902 (NCT05805956), the combination of trastuzumab and the HER2 TKI tucatinib (NCT04579380), and novel antibody–drug conjugatesA166 (NCT03602079) and zanidatamab zovodotin (NCT03821233).

#### 8.1.5. Multikinase Inhibitors

Regorafenib is an oral multikinase inhibitor targeting angiogenic kinases such as VEGFR, FGFR, and PDGFR, as well as mutant oncogenic kinases such as c-kit receptor tyrosine kinase (KIT), RET, and BRAF [162]. Several multicenter clinical trials were conducted to assess the efficacy of regorafenib for chemotherapy-refractory patients with BTC, showing that it significantly improved PFS and tumor control in patients in the second- or third-line setting [163,164]. The efficacy of regorafenib in combination with avelumab, a PD-L1 inhibitor, was also investigated in a phase II trial (NCT03475953) for BTC patients refractory to prior chemotherapy [165]. The trial reported an ORR of 13.8%, median PFS of 2.5 months, and median OS of 11.9 months. Other ongoing clinical trials are assessing the effectiveness of combining durvalumab plus regorafenib (NCT04781192) and cadonilimab plus regorafenib (NCT06335927) in patients with advanced and metastatic biliary tract cancers.

Lenvatinib is another oral multikinase inhibitor targeting VEGFR, FGFR, PDGFR, RET, and KIT that demonstrated antitumor activity in BTC, with a tolerable safety profile, in a multicenter phase II trial (NCT02579616) [166]. Combination therapy with lenvatinib and PD-1 (pembrolizumab/tislelizumab/sintilimab/camrelizumab/toripalimab) inhibitors have also been investigated, showing promising results [167,168,169]. Several clinical trials are also currently ongoing, evaluating the safety and efficacy of lenvatinib plus tislelizumab combined with gemcitabine and cisplatin (GPLET) (NCT05532059), lenvatinib plus sintilimab (NCT05010681), lenvatinib plus paclitaxel (NCT05170438), lenvatinib plus adebrelimab combined with gemcitabine and cisplatin chemotherapy (NCT06298968).

### 8.2. Immunotherapy

Next-generation sequencing and single-cell RNA sequencing (scRNA seq) of CCA have allowed researchers to identify novel immune subsets and pathways related to the immune system and the tumor microenvironment, paving the way to the application of immunotherapy in CCA management [39,170].

In recent times, immune checkpoint inhibitors, particularly those that block programmed death 1 and its ligand (PD1/PD-L1), have therefore emerged as promising strategies against a variety of cancers and are being increasingly integrated into the therapeutic landscape of CCA. A growing body of research supports that the use of PD1/PD-L1 monoclonal antibodies in conjunction with chemotherapy may significantly improve patient outcomes [171].

The results of the TOPAZ-1 trial (NCT03875235) in this way have opened new possibilities in the management of chemo-refractory advanced BTCs. The addition of durvalumab, a PDL-1 inhibitor, to the GEMCIS regimen has been proposed to be the first-line therapy in BTCs, showing improved OS rates. Updated overall survival and safety data from TOPAZ-1 continue to support this regimen as a standard of care for people with untreated, advanced biliary tract cancer [172].

Several ongoing clinical trials are currently further investigating the efficacy of durvalumab, especially in combination with olaparib (NCT06441747), durvalumab plus tremelimumab after an initial Standard of Care Specific Internal Radiotherapy (SIRT) in patients suffering from non-resectable intrahepatic BTCs (IMMUWHY phase II clinical trial, NCT04238637).

Toripalimab is another anti-PD-1 monoclonal antibody that has shown benefits in several cancers. In a phase II trial (NCT03951597), toripalimab was combined with lenvatinib and gemcitabine plus oxaliplatin (GEMOX) chemotherapy as first-line treatment to assess if PD-1 inhibitors can provide benefit also in iCCA patients. Data demonstrated high efficacy, controllable AEs, and feasibility of this triple-combination therapy for iCCA, which should be further explored in future prospective trials. To that end, a phase III study has been developed to confirm the high efficacy of this combination therapy in patients with advanced iCCA (NCT05342194) [173].

Camrelizumab is a humanized PD-1 IgG4 monoclonal antibody that has shown success in lymphoma, HCC, and lung cancer, and is beginning to be tested in CCA. In a phase II, multicenter trial (NCT03092895), advanced BTC (84.8% iCCA) patients received camrelizumab plus oxaliplatin-based chemotherapy, resulting in an overall ORR of 16.3%, a median PFS of 5.3 months, and a median OS of 12.4 months. Due to its favorable side effect profile and moderate efficacy, camrelizumab remains a drug of interest in supplementing standard chemotherapy [174]. A phase II clinical study (NCT04454905) is evaluating the safety, tolerance, and efficacy of camrelizumab in combination with apatinib in patients with advanced iCCA.

Combining CTLA-4 and PD-1 inhibitors could have a synergistic antitumor effect, resulting in enhanced activity and increased infiltration of TILs, as well as a decrease in Tregs in the CCA microenvironment.

In a multicenter phase II study involving 39 cases of metastatic BTC (16 iCCA, 10 eCCA, 13 gallbladder cancer), the combination of nivolumab (anti-PD-1 inhibitor) and ipilimumab (anti-CTLA-4 inhibitor) was investigated. An ORR of 23% was observed in 9 of 39 patients, with a disease control rate (DCR) of 44%. The median PFS was 2.9 months, with a median OS of 5.7 months. Immune-related toxic events were reported in 49% of patients, with 15% experiencing grade 3–4 events [175]. Hence, combination therapy with nivolumab and ipilimumab showed a substantial response in advanced iCCA cases. In this regard, a new clinical trial (NCT05921760) is evaluating the efficacy of combining ivosidenib, nivolumab, and ipilimumab in previously treated patients with non-resectable or metastatic IDH1 mutant CCA.

Another phase II trial (NCT04941287) investigates the effect of combining two immune therapies, atezolizumab (PDL-1 inhibitor) and CDX-1127 (varlilumab, an anti-CD27 antibody), with or without cobimetinib, a kinase inhibitor, in treating patients with unresectable biliary tract cancer. Immunotherapy with monoclonal antibodies, such as atezolizumab, may help the body’s immune system attack the cancer, and may interfere with the ability of tumor cells to grow and spread. Varlilumab is an immune agonist antibody that may further strengthen the immune system’s attack on the cancer.

Vaccination with tumor antigens appears to offer a potential opportunity to activate the immune system and prevent the development of an immunosuppressive tumor milieu. In this context, chimeric antigen receptor (CAR) T cell therapy has emerged as a promising immunotherapeutic approach for cancer treatment.

Currently, a phase II clinical trial (NCT03633773) is evaluating the therapeutic potential of MUC-1 CAR T cells to boost immunity in patients with CCA [176]. Enhancement of CAR T cell activity against CCA has been attempted by simultaneous knockdown of six inhibitory membrane proteins and this exhibited strong immunity against CCA and long-term efficacy both in vitro and in vivo [177]. A phase I clinical study (NCT06010862) is currently evaluating the safety and tolerability of CAR T in patients with carcino-embryonic antigen (CEA)-positive advanced/metastatic solid tumors.

In addition, some clinical trials are evaluating the effectiveness of T cell-based adoptive immunotherapy by reinfusing autologous tumor-specific T cells that had previously been expanded ex vivo into patients with CCA (NCT03820310, NCT06196658) [178]. In CCA, TIL therapy could be particularly valuable due to the immunosuppressive tumor microenvironment, which limits the effectiveness of the immune response. Early research suggests that expanding tumor-reactive T cells may enhance immune responses against CCA when used alongside immune checkpoint inhibitors that block inhibitory signals like PD-1/PD-L1 [179]. 

## 9. Conclusions

Next-generation sequencing (NGS) has improved the understanding of CCA tumor biology by uncovering the complex and diverse genomic landscape of this disease and trying to identify and classify CCA subtypes. However, their relevance is limited due to the heterogeneity of the results that still require them to be compared, unified, and clearly defined in a larger court of patients.

New fields in cancer biology have been also investigated in CCA, but further studies are necessary to better understand their involvement in tumor progression. For example, cellular senescence has become a hot spot in oncological research because of its potential as a therapeutic target. Induction of senescence followed by the selective removal of senescent cells (senolysis) has emerged as a promising strategy to overcome drug resistance and improve clinical outcome of certain cancer patients [64]. Unfortunately, till now, research addressing the utility of this approach for treating CCA is lacking.

Currently, the best treatment option for CCA remains the surgical resection; however, improvements in CCA genomic profiling have allowed researchers to develop molecularly targeted therapies which may be more successful than the ineffective gemcitabine-based chemotherapy. However, additional studies are required to improve the use of these targeted treatments, investigate combination approaches, and overcome drug resistance. In addition, as the tumor–stroma crosstalk facilitates tumor growth, immune evasion, metastasis, and resistance to therapy, new strategies addressing the TME should be developed.

## Figures and Tables

**Table 1:** Distinctive features of SD- and LD-iCCA subtypes.

	Small Duct Type	Large Duct Type
Main location	Peripheral hepatic parenchyma	Proximal to hepatic hilar region
Risk factors	Chronic viral hepatitis (hepatitis B and hepatitis C), cirrhosis (regardless of the cause), alcoholic liver disease, metabolic syndrome, diabetes mellitus, obesity, and non-alcoholic fatty liver disease (NAFLD)/non-alcoholic steatohepatitis (NASH)	Liver fluke infections (*Clonorchis sinensis* and *Opisthorchis viverrini*), primary sclerosing cholangitis (PSC), and hepatolithiasis
Macroscopic features	MF pattern	PI, PI + MF pattern
Mucin production	Non-mucin-secreting glands	Mucin-secreting glands
Tumor border	Expansive or pushing, rarely infiltrative	Infiltrative
Genetic background	IDH1/2 mutations, FGFR fusions	KRAS and SMAD4 mutations
Markers	CD56 (NCAM), C-reactive protein, N-cadherin, BAP1 (loss)	MUC5AC, MUC6, S100P, MMP7, SMAD4 (loss)
Immune profile pattern	Low levels of total TILs	Higher levels of total TILs
Outcome	Favorable	Poor

**Table 2:** Biological features of S1–S4 subtypes.

	S1	S2	S3	S4
Gene mutations	IDH1/2	TP53	KRAS	FGFR2/BAP1
Activated pathways	TGF-β, ErbB, and mTOR signaling	Cell cycle/DNA replication pathways GBP4 demethylation	High tumor immune variability Carbohydrate metabolism Cytoskeleton regulation/ECM interaction GBP4 demethylation	Oxidative phosphorylation
Outcome	Good survival	Poor survival	Poor survival	Good survival
DNA methylation profile	Global methylation loss	Lowest methylation level	Global methylation loss	Methylation level comparable to controls
Possible therapeutic approach	IDH inhibitors	Immunotherapy	Immunotherapy	FGFR inhibitors

**Table 3:** Dysbiosis in CCA.

Disease	Microorganisms	Location	References
CCA	Enrichment in *Muribaculaceae unclassified*, *Lachnospiraceae NK4A136 group*, *Escherichia Shigella*, *Klebsiella*, *Lactobacillus*, *Akkermansia*, *Muribaculum*	Gut	[122]
CCA	Enrichment in *Burkholderia-Caballeronia-Paraburkholderia*, *Faecalibacterium*, and *Ruminococcus_1*	Gut	[118]
iCCA	Enrichment in *Lactobacillus*, *Actinomyces*, *Peptostreptococcaceae*, and *Alloscardovia*	Gut	[123]
CCA	Enrichment in *Collinsella*, *Eisenbergiella*, *Anaerostipes*, *Paraprevotella*, *Parasutterella*, and *Verrucomicrobia*	Gut	[124]
iCCA	Differences in *Bacteroides*, *Faecalibacterium*, *Roseburia*, *Escherichia-Shigella*, *Subdoligranulum*, and *Prevotella_9*	Gut	[125]
iCCA	Enriched in *Firmicutes Actinobacteria* *Bacteroides* and *Acidobacteriota*	Tumor microbiome	[126]

**Table 4:** Prevalent gene mutations in CCAs.

Primary Site	Genetic Alterations	Rate
iCCA	FGFR2	7–14%
	IDH1	20–29%
	BRAF	3–7%
pCCA and dCCA	KRAS	37–57%
all BTC	ERBB2	6–15%
	RET	0–5%
	NTRK	0.2–0.7%
	MSI-high	2.2–3.2%
